# Partnership for International Development: Finland–Argentina Conference on Circular Economy and Bioeconomy with Emphasis on Food Sovereignty and Sustainability

**DOI:** 10.3390/ijerph19031773

**Published:** 2022-02-04

**Authors:** Dele Raheem, Arnaldo T. Soltermann, Laura Virginia Tamiozzo, Ariel Cogo, Leena Favén, Noor Jahan Punam, Claudio R. Sarmiento, Egidija Rainosalo, Franco Picco, Federico Morla, Armando Nilson, Anna Stammler-Gossmann

**Affiliations:** 1 Arctic Centre, University of Lapland, 96101 Rovaniemi, Finland; noorpunam@ulapland.fi (N.J.P.); anna.stammler-gossmann@ulapland.fi (A.S.-G.); 2 Department of Chemistry, Universidad Nacional de Río Cuarto, Río Cuarto 5800, CP, Argentina; csarmiento@exa.unrc.edu.ar (C.R.S.); fmorla@ayv.unrc.edu.ar (F.M.); anilson@ayv.unrc.edu.ar (A.N.); 3 INTA AER Rio Cuarto, National Institute of Agriculture Technology, Río Cuarto 5800, CP, Argentina; lauravtamiozzo@gmail.com; 4 INTA Lujan, CIAP (Swine Activities Information Center), Lujan 6700, CP, Argentina; agro@unr.edu.ar; 5 RDI Chemistry and Bioeconomy, Centria University of Applied Sciences, 67100 Kokkola, Finland; Leena.Faven@centria.fi (L.F.); egidija.rainosalo@centria.fi (E.R.); 6 Cooperative Initia Limited, Río Cuarto 5800, CP, Argentina; piccofranco@hotmail.com

**Keywords:** circular bioeconomy, social–regional development, food security, social goods, cooperative capital, digital technology, law, natural products

## Abstract

A joint collaboration between the Cuarto region of Argentina championed by the National University of Rio Cuarto and the Arctic Centre of the University of Lapland, Finland organised a conference on several topics that are related to food sovereignty, sustainability, circular economy and bioeconomy. The efficient utilisation of natural resources in both regions is an important theme in meeting the sustainable development goals agenda. Hence, this partnership between the partner institutions will lead to the cocreation of knowledge. The topics were multidisciplinary, and the discussion focussed on research and teaching opportunities for institutions in both countries. The experts from both countries will continue to engage on the possibility of promoting the research agenda in these important areas.

## 1. Introduction

The collaborative initiative between the North and the Global South is fostered by the University Partnership for International Development (UniPID). UniPID is a network of Finnish universities aiming to advance universities’ global responsibility and strengthen their response to global challenges. UniPID offers a variety of support services for interdisciplinary studies, research and societal impacts of universities that are related to global development.

FinCEAL develops Finnish Science, Technology and Innovation Cooperation between Europe, Africa, Latin America and the Caribbean through networking. FinCEAL BRIDGES contribute towards strengthening on-renewab cooperation, while expanding the thematic focus to Agenda 2030 for Sustainable Development and the 17 sustainable development goals. This is in recognition of the need for cross-sector and cross-country collaboration in pursuit of all the goals by the year 2030.

Climate change mitigation and the sustainable development goals are part of the Finnish government’s program. Finland aspires to be carbon neutral by 2035 [1]. In order to achieve a fossil-free economy, the circular economy will be key, and it requires research-based knowledge. Food sovereignty and sustainability are innovative themes worth further research collaboration between the Arctic Centre, University of Lapland and the National University of Rio Cuarto. We aim to initiate a discussion on collaborative research partnerships between the Arctic Centre of the University of Lapland, Finland and the Universidad Nacional de Río Cuarto, Argentina (UNRC). The Arctic is sparsely populated, but it is an important part of the whole globe as it reflects the immediate effects of climate warming, long-range transport of contaminants, and the increase in human activities and migration; also, there are concerns regarding existing and emerging infections, such as zoonotic diseases. The Finnish bioeconomy strategy based on sustainability for a low-carbon and resource-efficient society is now in place, especially when we consider that Finland has the highest percentage of forest in Europe (over 75%) and that the bioeconomy accounts for 16% of the Finnish economy and one-quarter of its export [2]. A unique feature of the Arctic region, with a population of about 4.5 million people, is that it consists of several Indigenous peoples []. Argentina, as one of the five countries in the Southern Cone, has a population of 45.8 million [4]. The Southern Cone region has over 50% of its lands classified as having agricultural potential, with positive projections for 2050 that offer a basis for a strong bioeconomy that will contribute both to food security and energy objectives, and will offer social opportunities [5]. Biodiversity resources in the region are also significant, as it contains some of the world’s most important biodiversity hotspots. It is also important to note that the partnership synergy in the bioeconomy-led development between Finland and the Southern Cone region will be worthwhile in the future.

In this case report, we explore how food sovereignty can be better promoted amongst local people in remote areas such as the Arctic and in the Southern Cone which has big countries. The coproduction of knowledge between Finland and Argentina on the management of natural resources is important within the context of bioeconomy and circular paradigms from a multidisciplinary perspective. In the partnership, we hope to answer specific questions such as what circularity and bioeconomy mean exactly in these two contexts. The emphasis of this study was on the utilization of natural resources within the bioeconomy and circular economy paradigm. The participants highlighted the relevance of natural bioactive compounds in promoting health, food sovereignty, sustainability, digitalisation, value addition and food product development that can empower the local people in both regions. 

## 2. Theoretical Considerations

The circular economy concept has been defined in various ways. Kirchherr et al., 2017 reviewed 114 definitions and defined it as “*an economic system that is based on business models which replace the ‘end-of-life’ concept with reducing, alternatively reusing, recycling and recovering materials in production/distribution and consumption processes, thus operating at micro level (products, companies, consumers), meso level (eco-industrial parks) and macro level (city, region, nation and beyond), with the aim to accomplish sustainable development, which implies creating environmental quality, economic prosperity and social equity, to the benefit of current and future generations*” [6]. There are likely to be differences when an urban city in the Southern Cone region is compared to rural communities in the Arctic. The implementation of the strategy will be crucial, and cocreation of knowledge can help in this regard. It was noted that the circular economy will only remain as a technical tool, and will not bring about changes in the current unsustainable economic paradigm if our consumption culture does not change [7]. The European Union (EU) is promoting the concept of a circular economy, but it has been rightly observed that it was created mainly by policy makers, the business community, and practitioners, and it will be important to have a bottom-up approach that includes local communities at grassroot levels [8,9]. On the concept of circular economy, some authors have argued that conceptual limitations that support many economic and environmental benefits from circular policies and business models are not important for practitioners, and they suggest further empirical research rather than theoretical discussions [10,11]. It was observed that there are various inconsistencies in the body of literature regarding how the circular economy (CE) can serve as a tool for sustainable development, and there is an incomplete understanding of how its long-term effects differ from those of the “linear” economy [12]. As a relatively recent concept, there is still a strong necessity to build on the theoretical foundations of CE to avoid running the risk of lacking systemic validity and critical social relevance, which can effectively address the socioecological challenges of the 21st century [10]. Most of the discourse on spatial circularity have focussed on countries in the Global North, such as Japan, The Netherlands, Canada, USA, Belgium, etc. Our case report is addressing the gap in spatial circularity for rural communities in overlooked regions such as the Arctic and Global South.

At a recent World Bioeconomy Summit, the bioeconomy was defined as “the production, utilization, conservation, and regeneration of biological resources, including related knowledge, science, technology, and innovation, to provide sustainable solutions (information, products, processes and services) within and across all economic sectors and enable a transformation to a sustainable economy” [13]. The bioeconomy concept will be better promoted by other scientific–technological variables, as proposed by [14,15,16]. Such variables include the following: advances in research and development in the field of biological engineering and sciences, technologies of the fourth industrial revolution, science and technology of materials (e.g., nanotechnology) and digitalization (e.g., information and communication technologies (ICTs) and the Internet of Things (IoT). These are important transformative drivers to harness primary and residual biomass, i.e., agricultural and food waste, not only to increase their recycling opportunities or shorten supply chains, but also as an alternative raw material to produce fuels/energy, chemical substances, bioplastics and pharmaceutical products, etc. [17].

Food sovereignty is a broad concept focused on the rights of people, rather than corporations and market institutions, as the actors that the transnational social movement La Vía Campesina believes have come to dominate the global food system and control how and what kind of food is produced [18]. In transforming the current food system, it has been argued that food sovereignty that gives more control to local people can be one of the appropriate solutions. Food sovereignty principles operate with/at different scales (household to global), factors (policies to resources), and dimensions (equity to sustainability. The concept of food sovereignty has been proposed as one solution to food insecurity and the climate crisis, supported by the promotion of agroecological practices that simultaneously preserve diversity, enhance ecosystem service functions, reduce reliance on energy-intensive inputs, and link farmer knowledge with political mobilization [19,20,21,22].

## 3. Materials and Methods

Cocreation of knowledge between Finland and Argentina to seek better ways to manage natural resources was employed as a method. We adopted a type of collaborative autoethnography to study society and cocreate knowledge in the Arctic and South Cone regions during this unprecedented pandemic era [23]. Sustainability cocreation involves a combination of resources, knowledge and capabilities across multiple stakeholders [24], and it is related to sustainable learning, relationship management and the support of sustainable tools that will lead to improvements in the value chain, products and services [25]. As a precedent to this meeting between Finland and Argentina, the first encounter was a meeting held in Buenos Aires in October 2018 on the occasion of the International Seminar on Bioeconomy and development: opportunities through cooperation between the Nordic countries and the Southern Cone, organized by the Ministry of Science, Technology and Productive Innovation of the Argentine Nation. On that occasion, Dele Raheem and Arnaldo Soltermann had their first meeting. Subsequently and inspired by that initiative, the UNRC made progress in the search for these interactions with Nordic countries, and Arnaldo Sotlermann visited the University of Aarhus in Denmark and the University of Alnarp in Sweden. In 2019, UNRC received a visitor from Aarhus Denmark University on the occasion of the First Meeting of Bioeconomy and Circular Economy. From the knowledge of the existence of a second meeting and from subsequent efforts made, especially by Dele Raheem, a new meeting emerged, characterized by strong institutional participation from both parties and an important overall participation in the organization of the meeting in 2021, which was recognized as more integrated than the previous ones and with greater institutional commitment.

In early October 2021, there was a virtual event organised at the Arctic Centre of the University of Lapland with welcome remarks from the Counsellor for Education and Science Team Finland Knowledge, Argentina and the Director of International Relations, University of Lapland. Team Finland Knowledge connects academic institutions outside Europe towards better solutions to global challenges. The event was part of the Second regional meeting on circular economy and bioeconomy organised by the Argentine partners. Discussions on collaborative research partnerships between the Arctic Centre of the University of Lapland, Finland and the Universidad Nacional de Río Cuarto, Argentina, were intensified.

The need for collaboration with Argentine partners was a driving force in the design of topics delivered by the Finnish partners to coincide with the hybrid regional conference in Argentina, where many stakeholders in the field of bioeconomy were in attendance. There were 26 participants from Argentina and Finland, including 14 speakers from both countries who shared their thoughts for several hours throughout the day on several topics in both physical and virtual modes. As indicated, the topics discussed under “Results and Discussion” were transdisciplinary, with participants from academia, research agencies, the Ministry of Agriculture, farmers, cooperatives, extension workers and students. There were live, simultaneous interpretations from English to Spanish and vice versa. The physical and virtual event in a zoom meeting considered the geographical time difference between the two countries. We also utilized social media by engaging followers with hashtag #AC_UNRC on Twitter. PowerPoint slides of the speakers’ presentations and short comments on their topics were circulated with live commentaries from Twitter followers mainly from Finland and Argentina.

This is a case report intended to foster the partnership between Finland and Argentina. The event was hampered by the lack of physical interactions due to travel restrictions and some meanings might be lost in translations. Another limitation was that some of the project’s technical materials were only available in Spanish. However, we have kept in constant communication to advance our ideas. The virtual zoom meeting was recorded for future training and development opportunities by both partners. All the topics discussed at the event are presented in the next section.

## 4. Results and Discussion

The hybrid event resulted in topics that were actively discussed by participants, and they agreed to follow up on areas of mutual interests that will help to promote sustainable development goals. A snapshot of the oral presentations delivered by the expert speakers at the event are summarized in Table 1 below:

### 4.1. Promoting Circular Economy in the Food Sector through Digital Solutions: Perspectives from Finland

There is an urgent need to transform our food system for both individual and planetary health, and this was recognized by the recent UN Food System Summit Scientific Group, where they emphasized that food is central to people, the planet and the sustainable development goals (SDGs) [26,27]. Improving the performance of our food system will be crucial to reaching the sustainable development goals. The food system has been a digital laggard in comparison to other systems such as the health sector, but it is gradually catching up, with many upcoming start-ups [28]. The industrial food system has been estimated to require an expenditure of 10–15 calories to produce one calorie of food, contributing to 22% of greenhouse gas (GHG) emissions [29].

The manufacturing industry is transitioning from the era of mass production into the era of smart production, where physical production merges with the opportunities created by digitalisation into cyberphysical systems. From the Finnish perspective, the smart specialisation concept is designed to ensure that the Arctic natural environment is turned into an opportunity in the form of natural resource utilisation, from which new and innovative business areas and networks are emerging alongside and within the traditional industries [30]. Digitalisation can help to minimize the environmental impacts of food processing, and ultimately improve sustainability. In meeting the demand of local consumers, distributed and localized manufacturing are likely to make a difference. The adoption of food digitalisation will open up market accessibility for locally produced food products in local communities. Digitalisation will have major impacts on local food systems in the future. In order to innovate and bring benefits to local foods with high quality before they end up in the market, a consistent supply of these foods will be required. This will be best guaranteed with support to growers, processors and other stakeholders that are involved in the food value chain.

In Finland, Food Economy 4.0 as an ecosystem connects traditional and new actors with end users in new ways. The know-how of biomaterials, modular processes, robotics and digital technologies will create new international business opportunities, but they will also improve the competitiveness of domestic food [31]. The collation of data on what is happening at each stage can provide useful information to help artisan food producers at local levels. With accurate data used to inform, the value chain of local products will lead to opportunities to improve on processes at different stages. It will also be easier to share best practices and monitor food safety. With the latest technology that incorporates big data, there will be a drive to map and integrate data from across the whole food supply chain, including weather and remote sensing in agriculture, tracing where raw ingredients were sourced from, the nutritional content of foods, and tracking how food has been produced and handled. These innovative breakthroughs are making their way into future food systems via smart labels on food that can be scanned to reveal a whole host of information about a product, which allows consumers to differentiate between products on health and sustainability grounds [32].

### 4.2. Agroindustrial Waste as an Opportunity to Build Up a New Technology-Based Company: A Case of Circular Bioeconomy

Introduction to the second meeting: The chairman of the event, Arnaldo Soltermann, distinguished between bioeconomy and circular economy from the Argentinian perspective and its historical development in his introductory speech. In the Argentinian bioeconomy context, emphasis was on production based on biological resources, which includes processes and methods by which goods and services are provided in a sustainable way. The Argentinian strategy has three important aspects: (i) use of renewable biomass and efficient processes to achieve sustainable production (ii) use of converging technologies that includes biotechnology, nanotechnology and digital technologies and (iii) integration between applications such as agriculture, health and industry. Global challenges such as climate change and the use of non-renewable energy sources such as fossil fuels were strongly considered. In the 1980s, Argentinian technical efforts were intensified in the use of genetically modified organism (GMO), direct seeding, biotechnology, new seeds, biofuel, bio alcohol, organic and agroecological production and those developments are today important in the economy of Argentina. The circular economy as a strategy aims to reduce the input of raw materials as well as the output of generated waste. It raises important challenges such as moving away from fossil fuels to renewable fuels with further discussion on the role of finance and economic performance. In this context, it will be important to note that not all bioeconomy development is part of a circular economy. Some important examples in Argentina are the first-generation bio-fuels like bio alcohol and biodiesel from corn or oilseeds. They are clearly part of bioeconomy but it is very difficult to consider as part of a circular economy.

According to Georgescu-Roegen [33], the economy is an extension of biological evolution and from the thermodynamic point of view any renewable energy source can exist. Then, the economy is a calculated global transformation activity, and the damage that can be caused should be well-calculated while maximizing benefits, which may sometimes be contrary to economic revenues.

In 1999, the Development Laboratory was established (Chemistry Department of the Universidad Nacional de Rio Cuarto, Argentina) as an interdisciplinary research group, which began its work by analysing the technical and economic possibilities of the existence of an oleochemical development centre in the south of Cordoba (province), compatible with Mercosur and other markets as an incipient contribution to the circular bioeconomy concept.

During its evolution, the Development Laboratory also helped small cooperatives and artisans to produce healthy foods for the whole community. Some examples of those work are as follows: (A) The Agroindustry wastes: The oil industry in Argentina produces annually nearly 9 million tons of oil and 600,000 ton of wastes from nearly 20 Mha of oilseed [34]. Waste from vegetable oil or used cooking oil industries introduces waste storage and potential pollution problems. The productive use of waste could yield an important activity that would enhance the existing oil industry, while solving the environmental problem at the same time. The research process yielded advanced production technology, with high-added-value products (vitamin E for animal feed, fatty acids and derivatives such as liquid soap) used as feedstock oilseed industry waste [35,36].

Liquid soap is an important market product [37], and research on soap manufacturing at the university is undertaken as a social good for the community. We propose to aid poor community areas with vulnerable populations with cleaning products to reduce the burden of diseases that can otherwise have impact on health budgets of the public sector. The Development Laboratory obtained previous agreements in this regard from different government levels including the university. The other product defined as vitamin E is a tocopherol acetate, concentrated in a percentage greater than or equal to 30% by weight [38,39]. It is an intermediate product that can be used as input for several industries. As secondary products, we obtained fatty acid fractions and fertilizers in large amounts.

The industrial processes were supported by original, well-proven scientific and technological developments condensed in the business plan. A patent application is being developed. The plants designed are flexible, with an initial production of 120 tons/year of liquid soap and 50 tons of vitamin E. The projected plants are easily replicable and reproducible and require a relatively low initial investment, with excellent IRR and NVP values. Thus, as a spin off involving several non-university members, *Initia* (a workers’ cooperative) was formed as a technology-based company with a *sui generis* organizational formation based on the cooperative capital. This last point could be instrumental in developing countries to ensure circularity from generated side streams [40], and it is presented here with all its innovative character. As is expected, the company was created to cause a significant impact on the local economy and on the local university culture, where sometimes they are reluctant to participate in specific production processes.

(B) New technologies and social convergences:

Nowadays, there is a greater awareness of the need to know the origin of foods, as exemplified by the “Protected designation of origin” (PDO) regulation. The PDO regulation covers agricultural products and foodstuffs that are produced, processed, and prepared in a given geographical area using recognized know-how in this specific zone or region [41]. In order to improve quality of production, there are several cooperatives in the Rio Cuarto region that process cheese and marmalades and produce eggs and vegetables while considering and respecting animal welfare. All these activities focus on responsibility, equity, solidarity, and ecological practices within the circular economy.

Based on the activities of INTA and FERICAMBIO experiences, our laboratory supports the production of safe and quality foods, with elaborate manuals of good practices for the production of jams and cheese, and we demonstrate the superior quality of chicken eggs following the principles of production with animal welfare. We support agroecology and organic production of vegetables, developing and manufacturing them through INITIA pest biocontrollers for those products that do not exist in the market.

### 4.3. FERICAMBIO Fair for the Exchange of Knowledge and Products of Family Farming

The Southern Cone is a geographic and cultural region composed of the southernmost areas of South America. It covers Argentina, Chile and Uruguay. The region has over 50% of its lands classified as having agricultural potential, with positive projections for 2050 that offer a basis for a strong bioeconomy that will contribute both to food security and energy objectives, with important social opportunities. Biodiversity resources in the region are also significant, with some of the world’s most important biodiversity hotspots. Similarly, Finland has the highest percentage of forest in Europe, as indicated in the introduction. The Argentinian Ministry of Agriculture promotes artisan and organic products in the Cuarto region, as compiled by Queiroz et al. for two cities in the South Cone region, i.e., Río Cuarto (Córdoba, Argentina) and Seropédica (Rio de Janeiro, Brazil) [42]. Commercialisation of various products such as bakery products and the exchange of products are also encouraged. The ministry provides farmers with fungicide applications [43]. Since 2012, different experiences in food marketing have been carried out, mainly with agroecological vegetables that are obtained from the city’s garden producers, with the support of the PRO HUERTA program, for example, bags are made with vegetables during the production season in spring, summer, autumn and winter. In 2014, participation in the “Table of Articulation” was established, made up of educational institutions, nongovernmental organisations (NGOs) and family garden producers in view of the need to produce nuclear products from family agriculture, i.e., egg producers, fresh vegetable producers, free-range chickens, cheeses, honey, agroecological organic herbs and the purchase of perishable goods, baked goods, preserves, jams, honey, cheeses, basketry, leather handicrafts, liquors, wines, recipes of agroecological biopreparations and other family agriculture products.

During 2016, the fortnightly organization of the sale of fresh vegetables and basic food supplies with previous orders and at fairs was intensified. In addition, meetings were held to analyse and expand municipal ordinance No. 1273/06 for the marketing of food based on new social demands and alternatives for the production and marketing of family farming, accompanied by dissemination with simple brochures. For this reason, there is a need to strengthen the alternative channels of commercialization of family farming (FF) products that began to have greater demand and sale in the year 2016.

This is how “Fericambio” was born, an exchange fair made up of an integration of public and private organizations and institutions that make the alternative systems of production and/or commercialization of family farming products known [42] (p. 6). With the participation of more than 50 producers and exhibitors, it was organized by the Pro Huerta AER program of the INTA of Rio Cuarto together with the Undersecretariat of Social Development of the Government of Rio Cuarto and the Faculty of Agronomy and Veterinary Medicine of the UNRC, the Developing Laboratory, the ministry of the province and the ministry of the nation and FAA.

It should be remembered that Fericambio is an initiative in which numerous institutions and social organizations participate, where seeds, seedlings, aromatics, handicrafts, baked goods, canned goods, beverages, cheeses, wool and leather, among other agricultural products, are exhibited, exchanged and commercialized. In addition to raising awareness, informing, and offering the inhabitants of Rio Cuarto and the region, it serves as a dynamic for collective learning on the subject.

The beneficiaries from Fericambio are producer cooperatives, family farmers, stalls who attend fairs, individuals interested in obtaining products from family agriculture (AF), educational institutions, NGOs and consumers.

### 4.4. CIAP Activities and Digital Technologies

This project on digital technology received the highest award for Innovation in Agricultural Technologies (CITA) last year (2020), and the members were recognized by Governor Carlos Verna at the opening ceremony of ExpoTecno 2018, which reinforces the positioning of the province of La Pampa as an inescapable reference of current precision livestock.

Another project is known as “Mobile breeding center for the improvement of swine genetics of small and medium-sized producers in La Pampa”. Dean Abelardo Ferrán together with Sebastián Ramos were responsible for executing the project, which aims to improve the quality of the final product and the productive and socioeconomic indices of primary production, which positively impact the entire pig chain, improving the profitability of the sector. At a national level, pig reproductive biotechnologies are restricted to intensive production systems. On the other hand, this project seeks high-merit genetics that are accessible to small and medium producers. Innovative large-scale operations are also forthcoming with a more sustainable focus. Biogas production, farm management systems and optimization of their production facilities are indicated as the main focus points. As mentioned in the presentation, companies such as Pacuca, Qualitá and “The Good Pig”, amongst others, are already implementing these sustainable projects [43]. The presenter thanked the organizers for their invitation and above all for being interested in the actions carried out by the Swine Activities Information Center (CIAP). An experience that yielded coproduction of knowledge was developed between Argentina and Uruguay with the participation of public universities of both countries and the National Institute of Agricultural Technology (INTA), through technical representatives, teachers, researchers and extension workers. The mission is to contribute to the sustainable development of the pig agri-food (SAP) systems of Argentina and Uruguay, and this is where the relevance of this call lies, for the author. The presenter and their colleagues are personally convinced by CIAP that this transit has definitely contributed to the axes of food sovereignty and sustainability. “We are vehicles of a good that, because it is so abundant and has full accessibility today, is somewhat undervalued, as is information”.

The presenter believes that the development of linkage networks between technical researchers, extension workers and producers, but also consumers and other actors of the agri-food systems, will ultimately result in the strengthening of the aforementioned axes. Some concrete contributions through projects link agrotechnical schools in Argentina and Uruguay, spreading the use of free, public and free-access management systems of farms through information and communication technologies, the construction of an interactive map that references the various actors of the SAPs of the countries that are part of the space, the monthly production of reports with economic results of production models that become decision tools for producers and technicians, and the weekly publication of the summary of CIAP information that reaches numerous users. On a personal note, the presenter left with a reflection on his thoughts on natural resources and digital solutions despite the apparent distance between the two countries involved (Finland and Argentina), their levels of development, and the high coincidence that we can find in the digital tools to address our common problems.

### 4.5. Growth Opportunities in the Nordic Bioeconomy—Plant-Based Raw Materials for Health-Promoting Products

Leena Favén briefly introduced the current status of the bioeconomy in Finland. According to the Finnish Natural Resource Institute, the added value-added bioeconomy in 2019 was EUR 26 billion [44], and the turnover of the natural products sector was EUR 780 million in 2020 [44]. In 2021, the global demand for health-promoting products such as essential oils, nutraceuticals and antioxidants was estimated to be over USD 350 billion in total [45]. In Finland, biobased raw materials such as cultivated and wild plants as well as forest and food industry side streams have not been utilized to their full extent, and there is a great opportunity to refine high value-added health-promoting products such as functional food, food supplements, cosmetics and pharmaceutical products from these high-quality, Arctic, raw materials grown in a clean environment [46].

One of the research and development interests of Centria’s Chemistry and Bioeconomy team is the extraction and characterization of valuable compounds from biobased raw materials in order to enhance the development of industrial refining of high value-added biobased products. Biobased raw materials in Finland grow under long daylight hours and a clean environment, and they are often organic and of premium quality. The concentrations of polyphenols and antioxidant capacities have been analysed in order to characterize the potential premium quality of various biobased raw materials of Nordic berries, leaves and herbs.

Project ideas were presented for future learning and collaboration opportunities, such as:

- Comparative studies on plant-based health-promoting ingredients utilized in different parts of the world and countries (Argentina and Finland);
- How to meet global challenges in sustainable plant-based raw material availability in order to provide food for a growing and ageing population;
- How to work towards global standardization of methods.

### 4.6. Circular Solutions for Small Breweries

The brewing of alcoholic drinks is part of most cultures globally. Therefore, the potential circular solution for Finnish breweries might also be well-executed in Argentina and other countries.

Malt, water, hops and yeast are the main input ingredients in brewing. Additionally, breweries are both energy- and water-intensive [47]. Energy is needed for heating and boiling liquids, and cooling requires a substantial amount of cooling water. As only the liquid product is of interest, the process’s main by-products are brewers spent grain (BSG), spent hops/trub and spent yeast. BSG is the major by-product, representing around 85% of the total by-products generated [48]. Furthermore, 100 hL of beer would produce 2 tons of wet spent grain, which usually contains 10–20% of dry matter. Additionally, the fermentation process produces CO_2_ which in most cases is emitted into the air.

Implementing the circular economy principles to all streams would help small breweries to improve their economy and would have a positive environmental impact. The main challenges are associated with setting up an economically feasible value chain and finding high-value products. Many Finnish breweries are located in rural and peripheral areas and have small outputs, i.e., producing less than 1000 hL of beer per year. Thus, breweries, especially those who have no access to cost-effective transport, e.g., hubs, cannot benefit from the agglomeration effect, technology and business capacities that are available in cities or more segregated locations [49]. Furthermore, the biggest passion of entrepreneurs in small artisan breweries is quality and taste, with less emphasis on the efficiency of the brewing process and advanced handling of side and waste streams. Value chain organisers and orchestrators or matchmakers are needed to assist entrepreneurs in finding stakeholders in the ecosystem for the upgrade of by-products to higher-value products [50].

Finnish beer is produced from barley. Barley crop is rich in proteins, and dry BSG contains about 15% of crude protein. This fact is recognised by farmers, and if there are cattle farms close to breweries, the by-product is picked up by them. Such collection practices aid a potential reduction in biowaste handling costs, such as transportation and gate fees of biowaste acceptors. Other minor routes include biogas production and composting.

Thus, higher-value products that can overpower pre-treatment and transport costs would inspire entrepreneurs in the creation of the value chain for better utilisation of this by-product.

Centria University of Applied Sciences is assisting the brewing industry in the estimation of the technological routes and identification of potential partners for collaboration in BSG, as well as other by-products’ utilisation. The technology includes:

- Preservation of BSG as it is susceptible to microbial growth and spoilage;
- Separation and purification of protein fraction from BSG;
- Identification of other by-product sources to increase the scale of the technologies being developed.

We expect that our work can inspire industries in creating novel circular economy-inspired business cases and improving the resource efficiency of biobased materials. This work is part of the SYMBIOMA project, financed by the EU’s Interreg NPA Programme.

### 4.7. Circular Economy as a Tool to Achieve Sustainability: How and Where Can Law Intervene?

In this talk, the author summed up the law’s relevance in governing the circular economy. As a contributor from Finland in this event, she felt the necessity of highlighting Finnish initiatives to promote a circular economy. Since Finland is a member of the European Union, the European regulatory context was also discussed.

The use of the notion of circular economy almost gives it away to the study of economics. What role does law play in a matter of economics?

Linear economy is opposed to circular economy, and it does not follow the throwaway culture. Due to existing environmental crises caused by overexploitation of natural resources, the concept of circular economy appears to be promising to bridge the gap between environmental protection and economic development. Simply put, it is of interest to economists, environmentalists as well as policy makers, but law has not intervened in this area as much. However, when it comes to implementation, law is of essence, as it can support the sustainable development principle of international environmental law. It is a disputed principle, but it requires states to reconcile economic development with protection of the environment. Sustainable development is also identified as a general aim of a circular economy. In order to guide the transition to circular economy, the EU proposed action plan 2020 to close the loop by focusing on consumption changes and production behaviour through reuse and recycling, as well as waste management [51].

Relevant EU regulations [52,53,54,55,56,57,58] include: Directive 2018/851; Ecodesign Directive (Directive, 2009/125/EC); the Energy Labelling Regulation (Directive, 2017/1369); the Ecolabel Regulation (Directive 66/2010); the Green Public Procurement Directive (Directive, 2014/24/EU); and the Extended Producer Responsibility Articles 8 and 8a in the Waste Directive (as amended by Directive, 2018/851/EU). Amongst EU member states, Finland was the first to approve a road map to a circular economy in 2016.

Legal practices that are able to reflect more social planning types of theory might better facilitate a smoother and swifter transition towards the circular economy [59]. A system that sees sustainability only as an exception to the main rule is untenable in today’s society, where the environmental challenge is one of the most important problems that developed countries are tackling head on. On the contrary, Pihlajarinne and Ballardini proposed that the incentives for promoting sustainable innovation and sustainable business models should be embedded directly into the provisions conferring exclusive rights, to implement the idea of a “sustainable” lifespan as a core principle to consider while defining the scope of all the intellectual property rights [60].

In conclusion, a circular economy can be achieved by combining economics, law and policy in a multidisciplinary manner. Instead of having strict laws backed by sanction, there could be an ideology of soft laws through which companies adding to the promotion of the circular economy can be rewarded—for example, tax reduction for their businesses.

### 4.8. Contributions of Agroecology to Food Sovereignty

The processes of regenerating soil using compost and regenerative agriculture were highlighted in this presentation. The practiced agriculture includes livestock, and trees are also integrated in the farms symbiotically. The concept of regenerative agriculture, agroforestry, and the introduction of flowers to attract pollinating insects is a welcome development in the contribution of agroecology to food sovereignty. In an assessment of various grassroots initiatives in Latin America, it was revealed that the application of the agroecological paradigm can bring significant environmental, economic and political benefits to small farmers and rural communities as well as urban populations in the region [61].

Faced with the obvious signs of unsustainability presented by the predominant agricultural model, agroecology is proposed as an alternative agricultural model, dispensing with the use of agrochemicals and synthetic fertilizers while respecting at the same time the natural behaviour patterns of animals in terms of their space, food and health. In recent years, the existence of agroecology experiences has grown notably in Argentina, and in particular in the province of Córdoba. The last National Agricultural Census revealed that 5277 fields practice agroecology, organic agriculture or biodynamic agriculture (two alternative methods of pesticide-free production). Agroecology was sustained in the world by a group of farmers who resisted in the twentieth century the advance of chemical management as an agricultural paradigm, given the evidence of its environmental and socioeconomic impacts.

The concept of food sovereignty emerged as a response to the global food crisis that adds to the aforementioned environmental problems. In this sense, in 1996, civil society organizations such as Southern Peasant Movements and La Via Campesina proposed the concept of food sovereignty, trying to overcome the concept of food security proposed by the Food and Agriculture Organization (FAO). Food security was proposed by FAO as a state in which all people enjoy, in a timely and permanent way, physical, economic and social access to the food they need, in quantity and quality, for its adequate consumption and biological use, guaranteeing them a general welfare state that contributes to the achievement of their development [62], a concept that, as stated, was considered insufficient by the peasant organizations mentioned.

The concept of food sovereignty, for its part, is considered as a deepening of the term of food security, since it encompasses new dimensions with a strong sociocultural and political nature: it includes aspects such as access and use of productive assets, mainly land, water and biodiversity; the valorisation and improvement of peasant and indigenous productive systems; the optimization of the diversity of the ecological offer; the respect, rescue and improvement of ancestral agroecological practices; the preservation of peoples’ identities; the construction of new institutions for productive diversification and exchange with access to fair price markets; the conservation of genetic and ecological diversity, health equity, prohibiting the use of genetically modified organisms (GMOs); and the development of local facilities to satisfy basic needs in food and locally processed, preserved and distributed products, which are culturally in demand [63].

As can be seen, food sovereignty is not only concerned with the availability of food in quantity and quality, but also with local development, access to markets for both suppliers and consumers, for the forms and conditions in which they are produced. Food is key for the preservation and respect of local cultural practices. So, food sovereignty can be thought of as a proposal under construction towards a productive and political model that allows for satisfying the right to adequate food.

Agroecology, and its purpose of avoiding the use of inputs from chemicals, deploys a series of technical strategies that consist of trying to imitate the biological processes of natural ecosystems in a field. For this, the regeneration of the soils is prioritized through various techniques, and the recovery of biodiversity—trees, biological corridors, flower cords—is incorporated, the size of the cultivated plots is reduced, and a certain level of spontaneous plant species is tolerated, where a series of beneficial insects fulfil part of their biological cycles. In this way, agroecology manages to obtain satisfactory productive results and economic results that are usually higher than the averages of regional agriculture, because money is not spent on chemical inputs such as fertilizers, herbicides, fungicides, or insecticides, or on genetically modified seeds.

It can be thought, then, that agroecological production, due to its strategies and objectives, will contribute the most to the development of food sovereignty in these territories.

### 4.9. Food from Primary Productions

Maize or corn is one of the most important crops in the world, with an average annual production of 1127 million tons [64]. Only 25% is used as human food, 55% is for animal feed and nearly 20% is for bioethanol production in Argentina. Biofuel is regarded as part of the bioeconomy and not the circular economy. Argentina is the fifth world producer of maize, with a production of 33 million tons and an exportable balance of 23 million tons [65]. The added value of maize or corn for human consumption must be an objective of food companies in order to add value to primary corn production. The international prices increase from 10 to 100 times when going from corn seed to snacks. The dry grinding results in a product of five times more added value, and it looks interesting for small companies. The predominant types of corn are the variants of normal corn that were developed in laboratories with the aim of reducing crop losses; some are resistant to insects and others to herbicides. Some countries still do not accept these modifications, and argue for non-GMO products. Franco Pico explains the complete processes of the dry grinding of maize grains to the final products.

Food products containing maize do not have gluten but have a high percentage of starch (high energy), and they are used for various foods such as soups, bread, flour and polenta, snacks, pasta, breakfast cereals, brewing, semolina and bran for biscuits. Other important products in the corn production chain involve other sectors of the industry such as oil, packaging material, paper, textiles, pharmaceutical industries and biodegradable bags.

Finally, as an example of the potential of the dry milling technology, is it possible with an investment lower than USD 500,000 to process 100 tons of corn per day, generating work for more than 10 people and producing cereal for breakfast, alongside other products. In addition, some by-products remain, e.g., the germ of the corn and the shell which are used for animal feeding and to extract oil. In a nutshell, dry milling is a method of adding value to the corn without the necessity of going through protein transformation using animals, generating a fast and healthy economic food.

### 4.10. Primary Production of Soybeans

The traditional and agricultural processes in soybean production are related to the circular economy in Argentina. There are several by-products including biodiesel from the processing of soybeans. However, biofuel is not to be construed as circular economy-related but it is part of the bioeconomy. Argentina generates lots of income from the export of soybeans, but there are environmental concerns. For instance, in 2019, Argentina exported USD 3.47 billion worth of soybeans, making it the third largest exporter of soybeans in the world. Most of the soybean exports from Argentina go to China (USD 3.01 billion), Egypt (USD 214 million), the United States (USD 64.6 million), Vietnam (USD 40 million), and Russia (USD 32 million) [66]. The processing techniques involved in the primary production of soybeans have varied over the last 100 years. Currently, efforts are being made to reduce contamination that can reduce the production output with better techniques.

Soybean production is one of the most relevant activities in the Argentine economy. The soybean agroindustrial complex is organized with a marked export profile, based on the industrialization of primary grain production as exemplified in the Cuarto region. It is the main export chain in the country (close to 30% of the total exports in the last 5 years), surpassing the cereal chain and the automotive chain. Most of the soybean is used for milling. The industrialization of soybeans mainly comprises the production of oils, flours and, more recently, biodiesel. Of the total production of crude soybean oil, more than 60% is destined for export, the rest for biodiesel production and refining (both for domestic consumption and for other industries). The by-products of the oil industry such as protein flours are processed for the production of balanced food for animal consumption, 90% of which are destined for the foreign market. Thus, Argentina is the world’s leading exporter of oil and pellets. The crop began to be sown in the mid-1970s, and in less than 50 years it has had an unprecedented advance, with production increases of more than 80 times the initial one. The primary link in the chain involves a large number of producers with a heterogeneous composition. In particular, a large group stands out, accounting for more than 50% of production. It is representative of large-scale agriculture consisting of land leasing, equipment and machinery rental, use of process technologies such as direct seeding and annual double cropping, and input packages based on genetically modified seeds, herbicides and fertilizers. In addition, the increase in production is due to the increase in the area planted. This has occurred due to the substitution of other crops or livestock fields, as well as due to the agricultural advance on deforested lands or with lower productivity than those of the Pampean agroecosystems. The main challenges of primary soybean production are being able to decouple the increases in yields obtained from the environmental impact they generate. When analysing the sustainability of soybean production, the following critical points were detected: 1.—The soybean cropping system in Argentina is characterized by being extractive of nutrients from the soil, according to INTA data for every 40,000 tons of soy that leave the country, there are approximately 3576 tons of nutrients. 2.—Advances towards areas with soils more susceptible to degradation. The expansion of the agricultural frontier advanced many times in places such as native forests whose soils are not suitable for this type of crop but which are an important source of environmental goods and services. The literature indicates that the deforestation of native forest leads to losses of organic carbon in biomass and soil, which increase greenhouse gas emissions and deteriorate the natural sinks of this element in the long term [19,22]. 3.—The risk of contamination by pesticides occurs almost exclusively in current agricultural production systems. There is a direct, high, and positive relationship between the amount of cultivated land and the relative risk of pesticide contamination. However, in relation to active substances, the risks of contamination by pesticides have been reduced notably in the last five decades due to the generation of products of lower toxicity and persistence. Finally, it is important to point out that there are currently strategies that integrate agronomic technologies and practices that would make it possible to stop or reverse environmental damage and make more efficient use of resources and inputs. Examples that can be cited are good agricultural practices, crop rotations, genetic improvement (biotechnology), crop management with ecophysiological and ecological bases, precision agriculture, agroecology, integrated management of harmful organisms, and multiple crops.

### 4.11. Potential Applications of Stevia Extract in Poultry Production

It is often preferred to have poultry products that are raised in nature instead of on antibiotics. This offers a promising outlook for *Stevia rebaudiana* with great potential in the avian industry.

Some of the potential benefits of this plant in avian animals include it having no additives, antioxidants, antimicrobials, or immunostimulants in its production. It can be employed in chicks for broilers. The addition of stevia at 0, 0.5, 0.75 and 1% was tested in the diet of chicks. The results showed lower toxicity at nanomoles per ml in the plasma of chicks with increased concentration.

In avian production, the international and national regulations have diminished the use of Antibiotic Growth Promoters (AGP). These regulations and the requirement from avian consumers which prefer natural products have directed researchers to look for natural growth promoters (NGP) as additives. NGP can include numerous substances: prebiotics, probiotics, enzymes, antimicrobials, and phytogenics such as Stevia (*Stevia rebaudiana* Bertoni) (S), with different mechanisms of action enhancing gut health.

*Stevia rebaudiana* Bertoni is a perennial herb native to Paraguay and Brazil, and is known as a natural sweetener due to the stevioside and rebaudioside (steviol glycosides) present in the leaves and stems. Together with the sweetening effects, they have numerous properties which are less known, such as antioxidant, antimicrobial, and antitumoral properties. There has been some research using S stems, leaves or extracts from leaves applying various extraction methods, with different results in humans, laboratory and productive animals. However, the mechanisms involved in these beneficial effects are still little known. In different research, both in vitro and in vivo, it was found that S can enhance their productivity performance and have properties such as antioxidant, antimicrobial, antitumoral, antifungal properties, and positive effects on the immunologic system.

Our research team over the past 3 years, has been researching the addition of S extract liquid or solid on broiler chicken from 1 to 15 or 21 days old, on water or diets (0.5–1%) with good results [67]. S extract liquid generated a better conversion index (*p* ≤ 0.05) and low peroxidation levels on broilers received this phytogenic at both doses. Regarding immunologic variables, S extract liquid increased Fabricius bursae weight (1% S extract liquid) and increased IgA levels of both 15- or 21-day-old chickens (0.5–1%). With respect to gut health, S extract generates a better Villi Height (VH)/Crypt Deep (CD) Ratio (*p* ≤ 0.05), overall at 1%. Regarding the S solid assay, the result was similar to liquid extract. S (0.5–1%) increased gut health by a better VH/CD Ratio (*p* ≤ 0.05), and increased plasmatic cells (Ig A producers) and goblet cell number, with a high mucus layer on the gut. Conclusion: S extract liquid or solid (0.5–1%) enhances gut histomorphometric variables, increasing gut health, when added to broilers’ diets to broilers during the first 15 days of life. This was reflected in a better conversion index in all broilers receiving this phytobiotic.

### 4.12. Nordic–Argentinian Cooperation

The Arctic Centre at the University of Lapland has been undertaking long cooperation activities in the field of Arctic and Antarctic social studies with institutions in Argentina since 2006. In 2006, a colleague from the Universidad del Salvador (Buenos Aires) was invited to the conference panel “Livelihood, politics and environment in flux” (VI conference: Reconsidering Peripheries and Centers, Rovaniemi, organizer Anna Stammler-Gossmann). Since then, the circumpolar activities started to develop and demonstrated a great potential for mutual academic benefits, involving northern and southern perspectives in their research. Afterwards, we had a series of joint events on circumpolar sociocultural aspects.

Circumpolarity became a focus of the “The Arctic and the Antarctic come together” Circumpolar Art and Science seminar at the Arctic Centre in 2007 (Rovaniemi, organizer Anna Stammler-Gossmann). Invited Argentinian colleagues contributed with different geographical, cultural, and scientific perspectives on the ways in which people feel and think about their environment.

In 2007 the “Arctic and Antarctic International Journal on Sociocultural Issues” (based in Buenos Aires) was established, and the author was invited to the editorial board. In 2010, she participated at the “1st International Conference on Circumpolar North and South (Socio-economic and sociocultural studies) at the Universidad Nacional de Buenos Aires”. These activities have greatly contributed to the development of a network between Nordic and Argentinian colleagues in the field of social science and to the growing interest in circumpolar perspective.

The following events were organized in close cooperation with the colleagues from Argentina:

- 2012. “How do we see the sea? Multiple meanings of water”. A week of lectures and documentary films in November (Arctic Centre, Arktikum, organizer, Anna Stammler-Gossmann);
- 2016. Founding meeting of the directive and scientific committee of the International Circumpolar Observatory (ICO) in Buenos Aires and Ushuaia (Organizers: Universidad del Salvador and Foundation for High Studies on Antarctica and Extreme Environments (FAE, Argentina). Since then, conferences on circumpolar sociocultural studies organized by ICO have been taking place annually. The educational activities have been an important aspect of these collaborative activities. Currently, the members of ICO are elaborating on the International Postgraduate Programme in Circumpolarity under the lead of the Universidad del Salvador (Faculty of Social Sciences). The programme proposes to develop five intensive online courses in Arctic and Antarctic Studies.

In 2016 and 2017, she conducted fieldwork in Tierra del Fuego and Santa Cruz (Argentina) and made observations on issues of cow breeding. The production of meat became of particular interest to her in the context of comparative study. Environmental challenges and patterns of adaptive practices in this sector in Patagonia showed close similarity to the processes in the sector in her research sites in Finland and Siberia. In her current developing interest in food studies (Food on the move), she is focusing on foodways that create new economic and societal ties and change established foodscape environments. Food consumption goes beyond sustenance or taste, and in proposed research, she focuses on the affiliation of foods with mobilization of not only economic, but also socially and culturally coded resources.

It would be a pleasure for her to continue the collaboration, with a possibility for a closer expertise exchange and for developing a comparative study in this complex process of changing the food environment that takes place in a transnational field. After her visits in Argentina, a comparative perspective has also been integrated into her teaching activities within the Arctic Studies Programme at the University of Lapland.

The innovative ideas that are presented in this conference will help to advance development in both regions. The intersection of traditional knowledge with modern ecology could result in the generation of knowledge that is simultaneously deep and broad [21]. Ultimately, innovation environments, when put into practice, are also known to help the process of disseminating the culture of open innovation [68], which can have influence on business acceleration and increase the productivity of academic research through the development and application of research. While reflecting on this case report, the interconnection of various disciplines can lead to the cocreation of knowledge on sustainability in both regions, as exemplified in this report. Reflexive practices are crucial to transdisciplinary research and will involve multiple positions held by the researcher [69,70].

Transdisciplinary sustainability researchers often target interdisciplinary research that encourage the ability to speak and move with agility across disciplines and epistemologies while developing their core discipline and becoming grounded in specific methods [71,72]. For example, Sellberg observed that to limit the risk of the research process that involves transdisciplinary approaches becoming more of a consultancy project, a transdisciplinary researcher needs to make sure that the cocreation process has sufficient space for learning, exploration and reflection, [73]. It is well-established that transdisciplinary researchers have to meet the demands of achieving ‘Triple S’—Scientific rigour, Societal relevance and the Self (who are the actors in the research practice) within this Triple-S heuristic. The Triple S outlines a relational space where these three aspects are interconnected, and to navigate this space involves engaging in networks and relationships with both human and nonhuman actors. The cocreated knowledge will be essential within the framework of a sustainable EU bioeconomy which is embarked upon by Finland. However, such framework must look beyond EU borders, promote sustainable trade conditions, promote social fairness, economic growth, and environmental protection [74]. Generally, a sustainable circular bioeconomy that we envisage in both the Arctic and South Cone regions should create economic opportunities for rural, coastal and urban communities through local biobased innovation, coupled with the integration of primary producers in value chains.

## 5. Food Sovereignty and Sustainability Problems in Rural Communities in Finland and Argentina

Food democracy is a process that puts people at the centre and gives them a voice and control over the transition towards more sustainable agri-food systems [75]. This is clearly related to food sovereignty, as highlighted in Section 2—“Theoretical considerations”. A growing body of evidence shows that the world today is not on track towards achieving SDG 2, i.e., ending hunger, food insecurity, and malnutrition in all its forms by 2030. Getting on track towards achieving SDG 2 will necessitate a move away from silo solutions towards more holistic, integrated solutions that address the food security and nutrition challenges both in countries and globally. The themes of circularity, bioeconomy and strengthening food sovereignty in rural communities will advance the sustainability goals. However, part of the criticism against the circular economy is that the study field is north-biased and dominated by researchers and organizations from developed, industrialized countries (e.g., USA, UK, Italy, Canada, The Netherlands, and Australia). Our case report is addressing the gap in spatial circularity for rural communities in overlooked regions such as the Arctic and Antarctic in the Global South. Baldy and Kruse suggests that while environmental and natural sciences are sufficiently addressed, social sciences, economics, and political sciences are generally overlooked [72]. The dimensions of sustainability are not mutually exclusive, and most studies address more than one dimension. Our approach engages multidisciplinary researchers and stakeholders in both countries to address the trade-offs between the different sustainability dimensions.

There are some common problems in remote rural areas in Finland and Argentina that endanger food sovereignty, for example there has been issues with migration of the younger generation moving to urban cities to seek employment. In the Argentine example, the promotion of soybeans as monoculture has environmental and human health implications, and it excludes small farmers, labourers, and indigenous people [76]. According to the current estimate (2021/22) by the USDA International Production Assessment Division, Argentina’s production of soybean is at 46.5 million metric tons and the land area used is 16.2 million hectares [77]. The overdependence on cultivating soyabeans has come at the expense of other crops grown by smallholder farmers. Biotech promoters claim the expansion of soybean cultivation as a measure of the successful adoption of the transgenic technology by farmers. It was observed that between 1998 and 2002, in one-quarter of farms in Argentina over a decade, soybean area increased by 126%, at the expense of dairy, maize, wheat and fruit production [78]. It can be argued that if smallholder farmers are able to productively cultivate monocrops such as soybeans without agrochemical inputs within the circular economy, bioeconomy paradigms will help to ensure environmental, social and economic sustainability. In this context, digital technologies can help to address the challenges towards sustainable, circular and biobased solutions. These solutions will be part of our future agenda in this international partnership.

It is pertinent to bear in mind that one social aspect that may influence how circular economy is practiced in Latin American countries could be related to different worldviews. Some authors have extensively discussed how the way different worldviews conceive “development” or “quality of life” depends on subjective and cultural aspects [79,80]. The indigenous groups such as the Saami in Finland and the more than thirty indigenous groups referred to as “Native Argentines” possess traditional knowledge that tend to be nature-dependent and can ensure more circularity with less waste. Economic activities are endangering the traditional livelihood of these indigenous peoples, such as mining in the Saami land in Finland and soybean land grabbers in Argentina. Therefore, it will be essential to consider when discussing circular economy and bioeconomy as sustainability-related topics how traditional ecological knowledge that has been practised by indigenous people can be incorporated into scientific knowledge. For instance, circular behaviours which are borne out of necessity already exist in lower-income countries in the Global South. A higher share of economic activities is related to repairing, reusing, or sorting waste, and thus, advantages for the transition to a circular economy may already exist. Circular economy needs to be integrated with collaborative, bottom-up, and innovative dynamics since it has a great potential for contributing to local and inclusive development efforts [81]. The COVID-19 pandemic in Argentina and other Latin American countries revealed significant shortcomings in the linear economy; the vulnerability of global value chains, the depletion of natural resources, and the exacerbation of social inequalities. The CE shows great potential if inclusive development is promoted in these regions. In line with the Finnish bioeconomy strategy to promote inclusiveness, competitive and sustainable bioeconomy solutions for global problems will be created in Finland, and new business will be generated both in the Finnish and international market [82]. Specifically, the goals of the bioeconomy strategy are: (i) a competitive operating environment for the bioeconomy, (ii) new business from the bioeconomy, (iii) a strong bioeconomy competence base, and (iv) accessibility and sustainability of biomasses.

The World Bank suggests that decision makers should aim to understand users and the technology, and should “engage in participatory iterative project design” to better engage small-scale farmers to clarify their specific needs concerning agricultural technologies [83]. According to the World Bank, collaborative needs cuts across the entire food and agricultural research process, from the conceptualization of a research program to the end users. When the soybean value chain in Argentina is considered, it is important to look beyond optimalisation efforts, not only on the production, but also on the whole value chain, which will ensure more locals are employed. In agricultural research for development, for example, priorities are often based on the digital needs of small-scale farmers with very limited resources. Lux research, which focuses on sustainable innovations that are commercially viable, highlights examples where digital technology can improve the food value chain, including ingredient informatics, cold-chain monitoring, automated food-quality inspection, and food traceability and transparency [83]. A more holistic approach of the food system, where every stage of the system has digital solutions, will be relevant towards transforming the food system.

When digitalisation within a local food system in Arctic Finnish Lapland is considered, there is a need for accurate data; the value chain of local products will lead to opportunities to improve on processes at different stages. Strengthening value addition to local food crops will help to reduce food miles and the transparency of where food comes from. It will also be easier to share best practices and monitor food safety. The collation of processing data parameters on what goes on at each stage can provide useful information to help artisan food producers at local levels that will be useful in the future [32].

## 6. Concluding Remarks and Outlook for the Future

The synergy in the bioeconomy- and circular economy-led development between Finland and Argentina will be worthwhile in the future. The research project at the Arctic Centre, University of Lapland explores cross-cutting issues from a multidisciplinary perspective related to the impacts of climate change, food security and a more sustainable means of food production, in order to minimize greenhouse gas emissions. Within the context of the food–energy–water nexus, digitalization and smart manufacturing as ways to produce food in a more efficient and sustainable way are relevant to food sovereignty. Food is known to be at the heart of the sustainable development goals, and the processes involved from farm to table will benefit from the knowledge coproduced with active research collaboration from the two regions. In our future outlook, we will be integrating the participation of indigenous peoples in both regions, integrating digital solutions that are appropriate for rural communities in Finland and Argentina, and the partnership will also look at the possibility of promoting the “Team Finland Knowledge” programme that has been created to strengthen cooperation between Finnish higher education institutions and the target regions and countries such as Argentina that are selected in the network. We plan to follow-up on the possibility of future collaboration in the near future by jointly developing grant applications that will be of mutual benefit to coproduce knowledge that can strengthen the circular economy and bioeconomy.

## Figures and Tables

**Table 1:** The title of topics presented and the author/speaker’s country.

Topic	Author	Country
1. Promoting circular economy in the food sector through digital solutions: Perspectives from Finland	DR	FINLAND
2. The perspectives of the circular economy applied to agro-industrial systems. The agro-industrial waste as an opportunity to build up a new technology-based company: A case of circular bioeconomy	AS	ARGENTINA
3. FERICAMBIO fair for the exchange of knowledge and products of Family Farming	LT	ARGENTINA
4. CIAP activities and digital technologies	AC	ARGENTINA
5. Growth opportunities in the Nordic Bio-economy—plant-based raw materials for health promoting products	LF	FINLAND
6. Circular solutions for small breweries	ER	FINLAND
7. Circular economy as a tool to achieve sustainability: How and where can law intervene?	NP	FINLAND
8. Contributions of Agroecology to Food Sovereignty	CS	ARGENTINA
9. Food from primary productions	FP	ARGENTINA
10. Primary production of soybeans	FM	ARGENTINA
11. Potential applications of stevia extract in poultry production	AN	ARGENTINA
12. Nordic- Argentinian Cooperation	AS	FINLAND

## Data Availability

Not applicable.

## Abbreviations

AC	Arctic Centre
AER	*Agencias de Extensión*, Extension Agencies
AF	*Agricultura Familiar*, Family Agriculture
AGP	Antibiotic Growth Promoters
BSG	Brewers Spent Grain
CD	Crypt Deep
CE	Circular Economy
CIAP	Swine Activities Information Center
CITA	Centre for Innovation in Agricultural Technologies
EU	European Union
FAA	*Federación Agraria Argentina*, Argentine Agrarian Federation
FERICAMBIO	*Feria de intercambio*, Trade fair
FinCEAL	Finnish Science, Technology and Innovation Cooperation between Europe, Africa, Latin America and the Caribbean
GHG	Greenhouse Gas
GMO	Genetically Modified Organism
ICO	International Circumpolar Observatory
INITIA	Workers Cooperative in Argentina
INTA	National Institute of Agricultural Technology
IoT	Internet of Things
IRR	Internal Rate of Return
NGO	Non-governmental organisation
NGP	Natural Growth Promoters
NPA	Northern Periphery and Arctic Program
NPV	Net Present Value
PRO HUERTA	National Governmental Program Fostering Food Safety of the Rural Poor
SAP	System Analysis Program for Pig agri-food
SDG	Sustainable development goal
SYMBIOMA	A 3-year project (2019–2022) financed by NPA and led by Centria University of Applied Science, Finland
TFK	Team Finland Knowledge
UniPID	Univers ity Partnership for International Development
UNRC	*Universidad Nacional de Rio Cuarto*
VH	Villi Height
